# Investigation of relative risk estimates from studies of the same population with contrasting response rates and designs

**DOI:** 10.1186/1471-2288-10-26

**Published:** 2010-04-01

**Authors:** Nicole M Mealing, Emily Banks, Louisa R Jorm, David G Steel, Mark S Clements, Kris D Rogers

**Affiliations:** 1 The Sax Institute, P.O. Box 123, Broadway NSW 2007, Australia; 2 School of Medicine, University of Western Sydney, Campbelltown Campus, Locked Bag 1797, Penrith South DC NSW 1797, Australia; 3 National Centre for Epidemiology and Population Health, Australian National University, ACT 0200, Australia; 4 Centre for Statistical and Survey Methodology, University of Wollongong, Northfields Avenue, North Wollongong NSW 2522, Australia

## Abstract

**Background:**

There is little empirical evidence regarding the generalisability of relative risk estimates from studies which have relatively low response rates or are of limited representativeness. The aim of this study was to investigate variation in exposure-outcome relationships in studies of the same population with different response rates and designs by comparing estimates from the 45 and Up Study, a population-based cohort study (self-administered postal questionnaire, response rate 18%), and the New South Wales Population Health Survey (PHS) (computer-assisted telephone interview, response rate ~60%).

**Methods:**

Logistic regression analysis of questionnaire data from 45 and Up Study participants (n = 101,812) and 2006/2007 PHS participants (n = 14,796) was used to calculate prevalence estimates and odds ratios (ORs) for comparable variables, adjusting for age, sex and remoteness. ORs were compared using Wald tests modelling each study separately, with and without sampling weights.

**Results:**

Prevalence of some outcomes (smoking, private health insurance, diabetes, hypertension, asthma) varied between the two studies. For highly comparable questionnaire items, exposure-outcome relationship patterns were almost identical between the studies and ORs for eight of the ten relationships examined did not differ significantly. For questionnaire items that were only moderately comparable, the nature of the observed relationships did not differ materially between the two studies, although many ORs differed significantly.

**Conclusions:**

These findings show that for a broad range of risk factors, two studies of the same population with varying response rate, sampling frame and mode of questionnaire administration yielded consistent estimates of exposure-outcome relationships. However, ORs varied between the studies where they did not use identical questionnaire items.

## Background

The aim of most epidemiological studies is to obtain estimates that can be generalised to a population of interest. For surveys concerned with disease prevalence, the main means to achieve this is to draw a sample that is sufficiently representative of the target population. However, few surveys have perfect response rates and any level of nonresponse can potentially lead to biased estimates of prevalence [1,2].

In contrast, much epidemiological practice is based around the principle that representativeness is not necessarily required for reliable estimates of relative risk based on internal comparisons within study populations [3-5]. Indeed, having a greater proportion of respondents in extreme categories compared to the population of interest may often be necessary, in order to yield sufficient information about specific exposure-outcome relationships [5]. A key issue is whether there is any nonresponse bias after conditioning on the covariates included in the analysis.

Cohort studies generally require more extensive data collection than one-off surveys, as well as the provision of identifying details and a long-term commitment to follow-up. While cohort studies often focus on selected population groups (e.g. occupational groups) [6,7] and have relatively high response rates within these groups, recent response rates to population-based cohort studies are usually below 50% [8-12]. Furthermore, cohort study participants are generally healthier and more health conscious than non-participants [3,13-16]. Concern is often expressed at the low response rates for cohort studies, or the selectiveness of the group under study, and the generalisability of their results [17].

Direct empirical data to support the assumption that internal comparisons remain reliable, despite low response rates or highly selected study groups, is lacking. Furthermore, concerns are also expressed that elements of study design, such as sampling methods and use of postal questionnaires versus interviews, may influence the observed relationships [18]. This paper investigates whether or not cross-sectional estimates of exposure-outcome relationships are affected by survey aspects (response rate, sampling frame and mode of questionnaire administration) or the wording of questionnaire items, by comparing estimates computed from two independent studies of the same target population with divergent response rates and different designs.

## Methods

### The 45 and Up Study

The 45 and Up Study is a population-based cohort study of more than 260,000 men and women aged 45 years and over in New South Wales (NSW), Australia [10]. Participants were randomly selected from the database that is used to administer the national universal health insurance scheme (Medicare Australia), which has almost complete coverage of the population. Equal numbers of males and females were selected for participants less than 80 years old. Individuals aged 80 years or over and residents in rural areas were oversampled by a factor of two, males aged 80 years or over were oversampled compared to females and all residents in remote areas were completely enumerated. Participants entered the study by completing a baseline postal questionnaire and providing written consent to have their health followed over time. The study questionnaire is available at http://www.45andUp.org.au. The survey was available only in English. The current overall response rate to the baseline questionnaire is estimated to be 17.9% [10]. The final analytic sample consisted of 44,851 men and 52,961 women joining the study up to July 2008 after excluding 125 respondents who had a missing Accessibility Remoteness Index of Australia (ARIA+) [19] score.

Post-stratification estimation weights were assigned to the 45 and Up baseline survey to adjust the sample to account for the differences in selection probabilities and response rates and give consistency with 2006 population estimates produced by the Australian Bureau of Statistics (ABS) [20]. The post-strata were formed according to sex (male or female), remoteness (major city, inner regional, outer regional or remote) and age (five year age groups from 45-85 years or ≥85 years).

### The NSW Population Health Survey

The NSW Population Health Survey (PHS) is an ongoing survey on the health of people in NSW using computer assisted telephone interviewing [21]. Independent samples of NSW households with private telephones are drawn each year using random digit dialling, and one person is randomly selected to participate in the survey. Informed consent was obtained from participants by their willingness to complete the telephone interview. The survey questionnaire is available at http://www.health.nsw.gov.au/publichealth/surveys/phs.asp. The survey is administered in 6 languages. In 2006 participants were asked all survey questions and in 2007 they were asked a random subset of the survey questions. We report analyses of data for 5,766 men and 9,030 women aged 45 years or over who responded to the 2006 (n = 5,480) or 2007 (n = 9,316) PHS, with response rates of 59.3% [22] and 63.6% [23] respectively.

Weights were assigned to each year of data to adjust for the differences in the probability of selection within the household, number of residential telephone connections to the house and the varying sampling fraction between each of the 8 NSW area health services to provide estimates that were representative of the NSW population [21]. These area health services can include several remoteness categories. Post-stratification weights were also assigned according to sex (male or female) and age (five year age groups from 45-85 years or 85-110 years) using 2005 and 2007 mid-year population statistics released by the ABS for each area health service [22,23]. After weighting, Indigenous people are slightly under-represented in the PHS sample, and Australian-born people slightly over-represented, compared to the overall NSW population [22,23].

### Questionnaire items

We obtained the original questionnaires from the 45 and Up Study and the 2006 and 2007 PHS and compared the wording of questions and response categories. We classified questionnaire items as highly comparable, moderately comparable or not comparable, based on whether the item was expected to yield identical, similar or non-comparable responses, respectively, for a given individual. Analyses focused on items considered highly or moderately comparable; items used in these analyses are compared in Additional file 1. All variables used in these analyses were derived from self-reported data except postcode (45 and Up Study only).

All analyses included all participants in both studies, unless otherwise stated. If one study only asked a sub-set of participants a question of interest then the same restriction was applied to the other study. Data are reported on falls in the past 12 months for participants aged 60 years and over, hysterectomy operation in females less than 70 years, mammography screening in the past two years for females less than 80 years and bowel screening in the past 5 years for all persons aged 50 years and over.

Questions on mammography screening and hysterectomy were only asked in the 2006 PHS and hypertension and bowel screening in the 2007 PHS.

### Highly comparable questionnaire items

Remoteness was determined using the mean ARIA+ score for the postcode of the participant's residential address and categorised as major city, inner regional, outer regional or remote, according to the Australian Institute of Health and Welfare [24].

Self reported height and weight were used to calculate participants' body mass index (BMI) as weight in kilograms divided by the square of height in meters. BMI was categorised as underweight (BMI < 18.5 kg/m^2^), normal range (BMI 18.5-24.9 kg/m^2^), overweight (BMI 25.0-29.9 kg/m^2^) or obese (BMI ≥ 30 kg/m^2^) according to the World Health Organisation [25].

Participants were classified as having hypertension, diabetes and/or asthma if they reported that these conditions had ever been diagnosed by a doctor (both studies) or at a hospital (PHS only). Only participants who answered version two of the 45 and Up baseline questionnaire were asked if they had ever been diagnosed with asthma (n = 65,522).

Indicator variables were constructed for being born in Australia, missing all natural teeth, speaking a language other than English at home, having fallen in the past 12 months, having private health insurance (excluding Medicare) and having a hysterectomy.

Daily fruit consumption was grouped into participants who don't eat fruit, participants who eat fruit but less than two serves per day, and participants who eat two or more serves per day.

An indicator for females who were breast screened in the past two years was ascertained from responses to ever having a mammogram and the year of (45 and Up Study) or time interval (PHS) since their last mammogram.

Psychological distress was evaluated using the Kessler (K10) measure [26] ascertained as the sum of responses for 10 questions. If a respondent answered nine of the 10 items then the missing item was imputed as the average of the other nine responses. If a respondent answered less than nine items their K10 score was set to missing. Participants with a K10 score of 22 or greater were assigned as having high/very high levels of psychological distress and those with a score less than 22 as having low/moderate levels of psychological distress [27].

### Moderately comparable questionnaire items

The wording of questions across the two studies differed for household income before tax (45 and Up Study included benefits, pensions and superannuation), bowel screening (screening tests varied by study) and current smoking status (45 and Up Study participants recorded whether they were 'regular smokers' currently without a definition for regular, whereas PHS participants recorded if they 'smoke daily' where smoking was defined to include cigarettes, cigars and pipes).

The response categories varied across the two studies for highest level of educational attainment (for these analyses similar categories were constructed) and self-rated health status (the PHS had an additional response category - for these analyses the categories "poor" and "very poor" on the PHS were combined).

### Analysis

Before analyses commenced, twenty exposure-outcome pairs were selected for inclusion in our analyses. These were selected on the basis of demonstrating relationships across a wide range of domains of research interest. This consisted of i) ten pairs where both the exposure and the outcome variables were highly comparable across the two studies; and ii) ten pairs where the exposure and/or outcome variables were only moderately comparable across the two studies.

Unweighted and survey weighted prevalence estimates with 95% confidence intervals (CI) were calculated for each study for all highly and moderately comparable variables used in the exposure-outcome relationship analyses. Odds ratios (ORs) were used to approximate relative risks and logistic regression analyses were used to calculate the 20 pre-determined exposure-outcome relationships for each study; separated into highly and moderately comparable ORs. In each case two sets of ORs were calculated; namely the crude OR and the OR adjusting for age, sex and remoteness since these were the sampling variables common to both surveys. Unweighted and weighted comparisons of these two types of ORs by study are presented in Additional files 2 and 3 respectively (45 and Up Study) and Additional files 4 and 5 respectively (PHS).

In the figures, the squares and lines represent each OR estimate and CI, with the area of each square being proportional to the sample size used for each estimate.

Wald chi-square statistics were computed to test for differences in the log odds ratios between the two surveys for each of the 20 exposure-outcome pairs and compared to a chi-square distribution with degrees of freedom equal to the number of categories minus one in the exposure variable. Each study was modelled separately and then the Wald statistics were calculated by combining the two sets of estimated parameters, variances and covariances. Analyses were conducted with and without using sampling weights. With survey data the Wald test can be unreliable if the degrees of freedom on the estimated covariance matrix are small [28]. In this case the samples were large and the designs relatively simple.

Analyses were carried out using SAS, version 9 [29]. This study has the approval of the University of New South Wales Ethics Committee and the NSW Population and Health Services Research Ethics Committee.

## Results

The distributions of social and demographic characteristics and of health risk factors and conditions in the two studies are shown in Tables 1 and 2, respectively. Younger persons and/or those living in major cities were under-represented in both surveys as were males in the PHS (the 45 and Up Study sample was stratified by sex). The prevalence confidence intervals were narrower on the 45 and Up Study compared to the PHS, because of the larger sample size.

**Table 1:** Social and demographic characteristics of the 45 and Up Study and the NSW PHS populations

	45 and Up Study			PHS		
Social and Demographics						
Characteristics ^a^	N	Crude % (95% CI)	Weighted ^b ^% (95% CI)	N	Crude % (95% CI)	Weighted ^c ^% (95% CI)
**Sex**						
Male	48851	48.0 (47.7, 48.3)	48.0 (47.6, 48.4)	5766	39.0 (38.2, 39.8)	48.4 (47.4, 49.5)
Female	52961	52.0 (51.7, 52.3)	52.0 (51.6, 52.4)	9030	61.0 (60.2, 61.8)	51.6 (50.5, 52.6)
						
**Age**						
45-49 years	12399	12.2 (12.0, 12.4)	18.9 (18.6, 19.2)	1803	12.2 (11.7, 12.7)	19.3 (18.3, 20.3)
50-54 years	16120	15.8 (15.6, 16.1)	17.1 (16.8, 17.4)	2043	13.8 (13.3, 14.4)	17.5 (16.6, 18.4)
55-59 years	17350	17.0 (16.8, 17.3)	15.9 (15.7, 16.2)	2188	14.8 (14.2, 15.4)	16.2 (15.4, 17.0)
60-64 years	15342	15.1 (14.8, 15.3)	12.6 (12.4, 12.8)	2252	15.2 (14.6, 15.8)	12.5 (11.9, 13.1)
65-69 years	12800	12.6 (12.4, 12.8)	10.1 (9.9, 10.3)	1939	13.1 (12.6, 13.6)	10.2 (9.6, 10.8)
70-74 years	9564	9.4 (9.2, 9.6)	8.3 (8.1, 8.5)	1710	11.6 (11.0, 12.1)	8.5 (8.0, 8.9)
75-79 years	7235	7.1 (6.9, 7.3)	7.3 (7.2, 7.5)	1490	10.1 (9.6, 10.6)	7.3 (6.9, 7.8)
80-84 years	7545	7.4 (7.2, 7.6)	5.4 (5.3, 5.6)	922	6.2 (5.8, 6.6)	5.9 (5.4, 6.3)
≥ 85 years	3457	3.4 (3.3, 3.5)	4.3 (4.1, 4.4)	449	3.0 (2.8, 3.3)	2.6 (2.3, 2.9)
						
**Remoteness (ARIA+)**						
Major City	44146	43.4 (43.1, 43.7)	69.1 (68.8, 69.4)	6718	45.4 (44.6, 46.2)	60.6 (59.6, 61.6)
Inner Regional	36640	36.0 (35.7, 36.3)	22.7 (22.5, 23.0)	4351	29.4 (28.7, 30.1)	24.4 (23.5, 25.2)
Outer Regional	18926	18.6 (18.4, 18.8)	7.6 (7.5, 7.7)	3086	20.9 (20.2, 21.5)	12.2 (11.6, 12.7)
Remote	2100	2.1 (2.0, 2.1)	0.6 (0.5, 0.6)	456	3.1 (2.8, 3.4)	1.3 (1.2, 1.5)
Missing	0	-	-	185	1.3 (1.1, 1.4)	1.5 (1.2, 1.8)
						
**Language other than English spoken at home**						
No	92230	90.6 (90.4, 90.8)	87.4 (87.1, 87.7)	13379	93.0 (92.6, 93.4)	89.5 (88.7, 90.3)
Yes	9580	9.4 (9.2, 9.6)	12.6 (12.3, 12.9)	986	6.9 (6.4, 7.3)	10.4 (9.6, 11.2)
Missing	2	0.0 (0.0, 0.0)	0.0 (0.0, 0.0)	17	0.1 (0.0, 0.2)	0.1 (0.0, 0.2)
						
**Country of Birth**						
Australia	75821	74.5 (74.2, 74.7)	70.8 (70.5, 71.1)	11332	76.6 (75.9, 77.3)	71.1 (70.1, 72.2)
Not Australia	24964	24.5 (24.3, 24.8)	28.2 (27.9, 28.6)	3433	23.2 (22.5, 23.9)	28.7 (27.7, 29.7)
Missing	1027	1.0 (0.9, 1.1)	1.0 (0.9, 1.0)	24	0.2 (0.1, 0.2)	0.2 (0.1, 0.2)
						
**Private Health Insurance**						
No	38300	37.6 (37.3, 37.9)	33.9 (33.6, 34.3)	6766	45.7 (44.9, 46.5)	41.5 (40.4, 42.5)
Yes	63508	62.4 (62.1, 62.7)	66.1 (65.7, 66.4)	7973	53.9 (53.1, 54.7)	58.2 (57.1, 59.3)
Missing	4	0.0 (0.0, 0.0)	0.0 (0.0, 0.0)	57	0.4 (0.3, 0.5)	0.3 (0.2, 0.4)

**Educational Attainment**						
No School Certificate	12385	12.2 (12.0, 12.4)	10.9 (10.7, 11.1)	2017	13.7 (13.1, 14.2)	11.7 (11.1, 12.3)
School Certificate	22608	22.2 (22.0, 22.5)	20.3 (20.0, 20.6)	3732	25.3 (24.6, 26.0)	20.6 (19.8, 21.4)
Trade/Certificate/Diploma	32289	31.7 (31.4, 32.0)	31.0 (30.6, 31.3)	1631	26.5 (25.8, 27.2)	29.5 (28.5, 30.5)
Higher School Certificate	9787	9.6 (9.4, 9.8)	10.1 (9.9, 10.3)	3902	11.1 (10.6, 11.6)	10.9 (10.2, 11.6)
Tertiary Qualification	22802	22.4 (22.1, 22.7)	25.9 (25.6, 26.3)	3096	21.0 (20.3, 21.7)	25.0 (24.0, 26.0)
Missing	1941	1.9 (1.8, 2.0)	1.8 (1.8, 1.9)	360	2.4 (2.2, 2.7)	2.3 (2.0, 2.6)
						
**Household Income**						
<$20,000 p.a	20633	20.3 (20.0, 20.5)	17.9 (17.6, 18.1)	4505	30.4 (29.7, 31.2)	22.2 (21.4, 22.9)
$20,000-$39,999 p.a	18386	18.1 (17.8, 18.3)	15.3 (15.1, 15.6)	3145	21.3 (20.6, 21.9)	20.3 (19.5, 21.1)
≥$40,000 p.a	39792	39.1 (38.8, 39.4)	44.6 (44.2, 44.9)	4768	32.2 (31.5, 33.0)	41.4 (40.3, 42.5)
Missing	23001	22.6 (22.3, 22.8)	22.2 (21.9, 22.5)	2378	16.1 (15.5, 16.7)	16.2 (15.4, 17.0)
						

ARIA+, Accessibility Remoteness Index of Australia; CI, Confidence Interval; p.a, per annum

^a ^Characteristics above the line-break are highly comparable across the two surveys. Characteristics below the line-break are moderately comparable across the two surveys. Weighted prevalence estimates for age, sex and remoteness differ across the two studies since the weighting schemes vary.

^b ^Weighted by age, sex and remoteness

^c ^Weighted by area health service, probability of selection in the household and the number of residential connections to the house

**Table 2:** Health risk factors and conditions of the 45 and Up Study and the NSW PHS populations

	45 and Up Study			PHS		
	
Variable ^a^	N	Crude % (95% CI)	Weighted ^b ^% (95% CI)	N	Crude % (95% CI)	Weighted ^c ^% (95% CI)
**Fruit Consumption**						
Don't eat fruit	6620	6.5 (6.4, 6.7)	6.5 (6.4, 6.7)	321	6.1 (5.5, 6.8)	6.1 (5.2, 6.9)
< 2 serves per day	31707	31.1 (30.9, 31.4)	31.0 (30.7, 31.4)	1719	32.8 (31.6, 34.1)	33.4 (31.8, 35.1)
≥ 2 serves per day	57620	56.6 (56.3, 56.9)	56.7 (56.3, 57.0)	3135	59.9 (58.5, 61.2)	59.4 (57.7, 61.2)
Missing	5865	5.8 (5.6, 5.9)	5.7 (5.6, 5.9)	61	1.2 (0.9, 1.5)	1.0 (0.7, 1.3)
						
**Teeth**						
Some/all natural teeth	88501	86.9 (86.7, 87.1)	88.0 (87.8, 88.2)	9321	86.7 (86.0, 87.3)	90.3 (89.7, 90.9)
No natural teeth	9953	9.8 (9.6, 10.0)	8.7 (8.5, 8.9)	1427	13.3 (12.6, 13.9)	9.7 (9.1, 10.3)
Missing	3358	3.3 (3.2, 3.4)	3.3 (3.2, 3.5)	6	0.1 (0.0, 0.1)	0.0 (0.0, 0.1)
						
**Body Mass Index**						
Underweight	1461	1.4 (1.4, 1.5)	1.5 (1.4, 1.6)	230	2.1 (1.9, 2.4)	1.9 (1.6, 2.3)
Normal Range	35219	34.6 (34.3, 34.9)	35.6 (35.2, 35.9)	4214	39.0 (38.1, 40.0)	38.4 (37.2, 39.6)
Overweight	37373	36.7 (36.4, 37.0)	36.6 (36.2, 36.9)	3774	35.0 (34.1, 35.9)	36.0 (34.8, 37.2)
Obese	20271	19.9 (19.7, 20.2)	19.2 (18.9, 19.5)	2205	20.4 (19.7, 21.2)	20.5 (19.4, 21.5)
Missing	7488	7.4 (7.2, 7.5)	7.1 (6.9, 7.3)	373	3.5 (3.1, 3.8)	3.2 (2.8, 3.7)
						
**Ever diagnosed with hypertension**						
No	72023	70.7 (70.5, 71.0)	72.1 (71.7, 72.4)	1189	52.1 (50.1, 54.2)	55.2 (52.6, 57.8)
Yes	25144	24.7 (24.4, 25.0)	22.9 (22.6, 23.2)	1086	47.6 (45.6, 49.7)	44.6 (42.0, 47.2)
Missing	4645	4.6 (4.4, 4.7)	5.0 (4.9, 5.2)	6	0.3 (0.1, 0.5)	0.2 (0.0, 0.4)
						
**Ever diagnosed with diabetes**						
No	88056	86.5 (86.3, 86.7)	86.6 (86.3, 86.8)	9450	88.4 (87.8, 89.0)	89.2 (88.4, 90.0)
Yes	9111	8.9 (8.8, 9.1)	8.4 (8.2, 8.6)	1202	11.2 (10.6, 11.8)	10.5 (9.7, 11.2)
Missing	4645	4.6 (4.4, 4.7)	5.0 (4.9, 5.2)	36	0.3 (0.2, 0.4)	0.3 (0.2, 0.4)
						
**Ever diagnosed with asthma**						
No	55034	84.0 (83.7, 84.3)	83.8 (83.5, 84.1)	8753	82.0 (81.3, 82.8)	83.2 (82.2, 84.1)
Yes	7653	11.7 (11.4, 11.9)	11.4 (11.1, 11.7)	1894	17.8 (17.0, 18.5)	16.7 (15.8, 17.6)
Missing	2835	4.3 (4.2, 4.5)	4.8 (4.6, 5.0)	22	0.2 (0.1, 0.3)	0.1 (0.1, 0.2)
						
**Fallen in the past 12 months**						
No	42038	75.1 (74.8, 75.5)	74.1 (73.7, 74.6)	2957	77.3 (76.0, 78.7)	77.5 (75.9, 79.1)
Yes	10757	19.2 (18.9, 19.6)	20.0 (19.6, 20.4)	856	22.4 (21.1, 23.7)	22.1 (20.6, 23.7)
Missing	49017	5.6 (5.4, 5.8)	5.8 (5.6, 6.1)	11	0.3 (0.1, 0.5)	0.4 (0.1, 0.6)
						
**Last Mammography Screening**						
Not within past 2 years	12622	26.2 (25.9, 26.6)	28.6 (28.1, 29.1)	1018	34.4 (32.7, 36.1)	36.7 (34.5, 39.0)
Within past 2 years	30056	62.5 (62.1, 62.9)	60.1 (59.6, 60.6)	1925	65.1 (63.4, 66.8)	62.8 (60.6, 65.1)
Missing	5413	11.3 (11.0, 11.5)	11.3 (11.0, 11.7)	15	0.5 (0.3, 0.8)	0.4 (0.2, 0.7)
						
**Hysterectomy**						
No	29487	73.9 (73.4, 74.3)	76.6 (76.1, 77.0)	1583	71.1 (69.2, 73.0)	74.2 (72.0, 76.4)
Yes	10431	26.1 (25.7, 26.6)	23.4 (23.0, 23.9)	630	28.3 (26.4, 30.2)	25.0 (22.8, 27.2)
Missing	0	-	-	13	0.6 (0.3, 0.9)	0.8 (0.3, 1.3)
						
**Psychological Distress**						
Low\Moderate	81542	80.1 (79.8, 80.3)	80.7 (80.4, 81.0)	9503	88.8 (88.2, 89.4)	88.9 (88.1, 89.7)
High\Very high	6663	6.5 (6.4, 6.7)	6.8 (6.6, 7.0)	1101	10.3 (9.7, 10.9)	10.2 (9.4, 11.0)
Missing	13607	13.4 (13.2, 13.6)	12.5 (12.2, 12.7)	100	0.9 (0.8, 1.1)	0.9 (0.7, 1.2)

**Current Smoker**						
No	93685	92.0 (91.9, 92.2)	91.9 (91.7, 92.1)	9468	87.9 (87.3, 88.6)	88.1 (87.3, 89.0)
Yes	7575	7.4 (7.3, 7.6)	7.6 (7.4, 7.8)	1292	12.0 (11.4, 12.6)	11.8 (11.0, 12.7)
Missing	552	0.5 (0.5, 0.6)	0.5 (0.5, 0.6)	7	0.1 (0.0, 0.1)	0.1 (0.0, 0.1)
						
**Last Bowel Screening**						
Not within past 5 years	49036	54.8 (54.5, 55.2)	55.9 (55.6, 56.3)	2497	53.5 (52.1, 54.9)	54.6 (52.7, 56.4)
Within past 5 years	35990	40.3 (39.9, 40.6)	39.2 (38.8, 39.6)	2098	45.0 (43.5, 46.4)	43.3 (41.5, 56.4)
Missing	4387	4.9 (4.8, 5.0)	4.9 (4.7, 5.0)	72	1.5 (1.2, 1.9)	2.2 (1.4, 2.9)
						
**Self-reported Health Status**						
Excellent	14920	14.7 (14.4, 14.9)	15.5 (15.2, 15.8)	2433	17.8 (17.2, 18.5)	18.1 (17.2, 18.9)
Very Good	36046	35.4 (35.1, 35.7)	35.4 (35.0, 35.7)	3815	28.0 (27.2, 28.7)	28.5 (27.5, 29.5)
Good	33178	32.6 (32.3, 32.9)	32.1 (31.7, 32.4)	3990	29.2 (28.5, 30.0)	29.8 (28.7, 30.8)
Fair	11878	11.7 (11.5, 11.9)	11.4 (11.2, 11.6)	2265	16.6 (16.0, 17.2)	16.0 (15.2, 16.8)
Poor	2090	2.1 (2.0, 2.1)	2.0 (1.9, 2.1)	863	6.3 (5.9, 6.7)	5.8 (5.3, 6.3)
Very Poor ^d^	N/A			239	1.8 (1.5, 2.0)	1.6 (1.3, 1.9)
Missing	3700	3.6 (3.5, 3.7)	3.7 (3.5, 3.8)	44	0.3 (0.2, 0.4)	0.3 (0.2, 0.4)

CI, Confidence Interval; NSW, New South Wales;

^a ^Variables above the line-break are highly comparable across the two surveys. Variables below the line-break are moderately comparable but similar across the two surveys

^b ^Weighted by age, sex and remoteness

^c ^Weighted by area health service, probability of selection in the household and the number of residential connections to the house

^d ^The category 'Very Poor' was only available on the NSW Population Health Survey

The weighted estimates of prevalence were similar across the two studies for variables such as age, sex, and remoteness (the variables used for weighting), country of birth, educational attainment, fruit consumption, body-mass-index and falls. However, the prevalence of speaking a language other than English at home and of holding private health insurance was higher in the 45 and Up Study compared to the PHS, while the prevalence of smoking, high/very high psychological distress, ever diagnosed with hypertension, ever diagnosed with diabetes and ever diagnosed with asthma was lower (Table 1; Table 2). The PHS tended to have less missing data than the 45 and Up Study, particularly for variables relating to mammography screening, K10 score and household income before tax. Prevalence estimates for self-rated health status varied across the two studies with the proportion who reported the lowest category of self-rated health status on the 45 and Up baseline questionnaire (i.e. "poor") being similar to the proportion who reported the lowest category on the PHS (i.e. "very poor")(Table 2).

**Figure 1:**
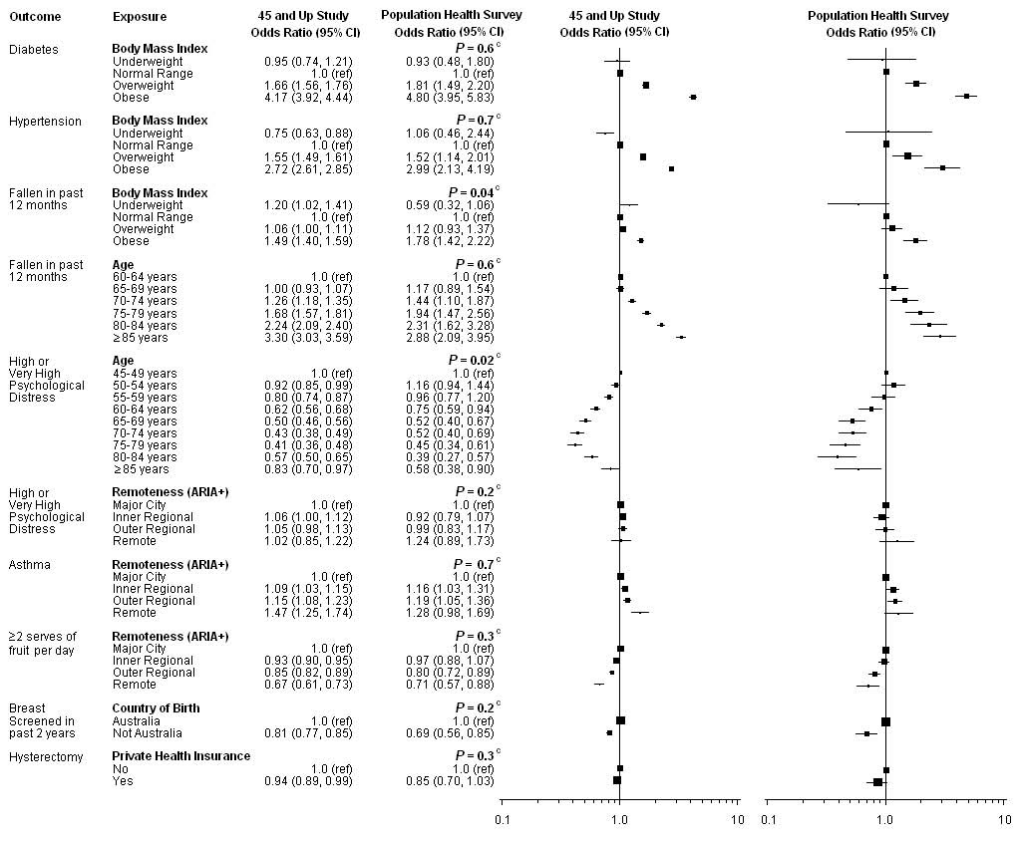
**Odds ratios ^a ^by study where the exposure and outcome variables were highly comparable across the 45 and Up Study and the NSW PHS ^b^**. ARIA+, Accessibility Remoteness Index of Australia; CI, Confidence Interval; NSW, New South Wales; PHS, Population Health Survey. ^a ^Adjusting for age, sex and remoteness. ^b ^Black squares represent ORs with area inversely proportional to the sample size contributing to the OR and the corresponding line represents the 95% confidence interval. ^c ^P-value from Wald chi-square test: testing for a difference between the two studies for the specific exposure-outcome pair

The ten exposure-outcome relationships where both the exposure and the outcome variables were highly comparable across the two studies are presented in Figure 1, with ORs adjusted for age, sex and remoteness. The observed relationships were virtually identical between the two studies. For 8 out of the 10 relationships there was no significant difference between the results from the different studies. There was borderline evidence of a difference in the risk of falling according to BMI across the two studies (Wald test *P *= 0.04) and minor heterogeneity in the relationship of age to high/very high psychological distress was observed (Wald test *P *= 0.02). Similar observations were seen when the ORs from these ten relationships were calculated using sampling weights (Additional file 6).

**Figure 2:**
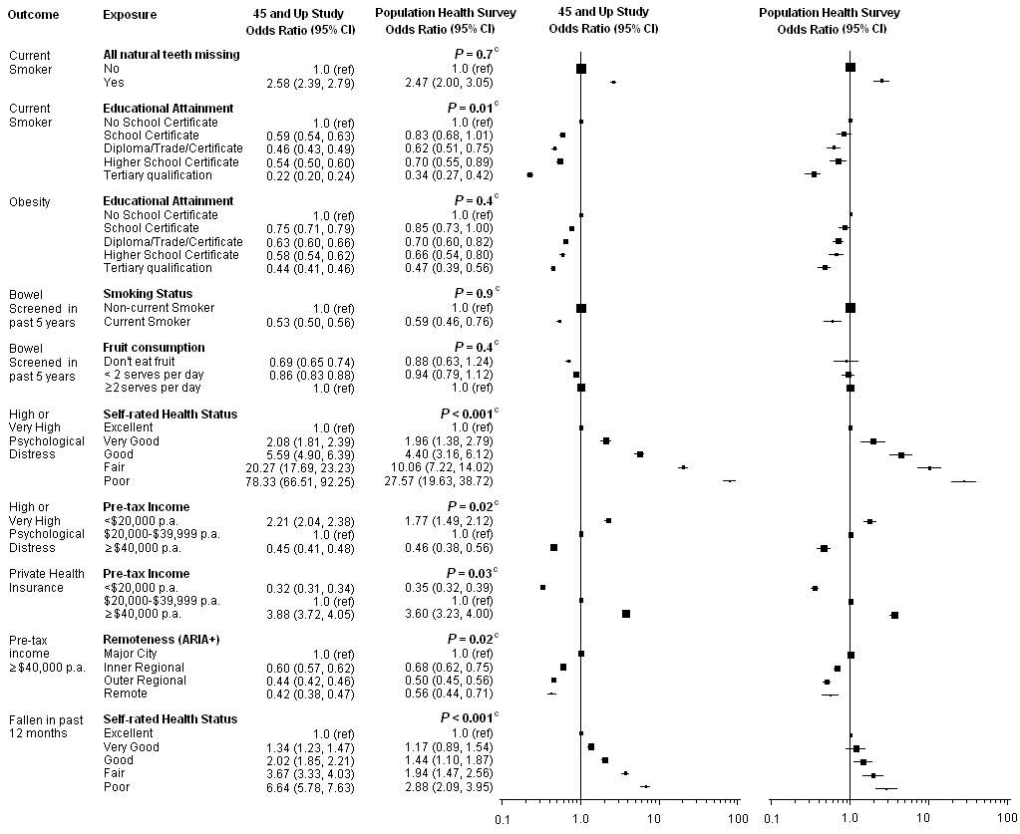
**Odds Ratios ^a ^by study where either the exposure or outcome or both variables were only moderately comparable across the 45 and Up Study and the NSW PHS ^b^**. ARIA+, Accessibility Remoteness Index of Australia; CI, Confidence Interval; NSW, New South Wales; p.a, per annum; PHS, Population Health Survey. ^a ^Adjusting for age, sex and remoteness. ^b ^Black squares represent ORs with area inversely proportional to the sample size contributing to the OR and the corresponding line represents the 95% confidence interval. ^c ^P-value from Wald chi-square test: testing for a difference between the two studies for the specific exposure-outcome pair

The ten exposure-outcome relationships where the exposure and/or outcome variables were only moderately comparable across the two studies are presented in Figure 2, with ORs adjusted for age, sex and remoteness. Each exposure-outcome pair had a similar relationship pattern for both studies and all OR estimates were in the same direction and of similar magnitude, except when self-rated health status was the exposure variable. The relationships did not differ significantly for 4 out of 10 of the exposure-outcome associations. Significant but relatively minor differences in ORs were observed for smoking and educational attainment and pre-tax income in relation to psychological distress, private health insurance and remoteness of residence. In spite of the similarity in the shape of the relationship, substantial heterogeneity and large differences in ORs were observed for relationships with self-rated health (where the PHS had an additional response category, "very poor"). Similar observations were seen when the ORs from these ten relationships were calculated using sampling weights (Additional file 7).

Following adjustment for age, sex and remoteness, additional weighting of the OR for age, sex and remoteness did not change any of the ORs from the 45 and Up Study materially (i.e. no changes were >10%) (Additional files 2, 3). This is because the weighting is determined by the variables used in the logistic regression. Weighting the PHS resulted in some changes to the ORs because not all variables used to determine the weighting (i.e. household size to account for the selection of a person from each selected household and the 8 area health services) were used in the logistic regression (Additional files 4, 5). Weighting did not change the general nature of the observed relationships.

## Discussion

Discussions around epidemiological methods often conclude that representativeness is not necessarily required for reliable estimates of relative risk based on internal comparisons within study populations [4]. By their nature, cohort studies tend not to be directly representative of the general population, however over time, their results have usually been shown to be both reproducible and generalisable to the larger population [6,7]. Miettinen explains that *"an empirical relation is not distorted by any manipulation of the distribution of the study base according to the elements in the occurrence relation - the determinant, the modifiers and/or confounders. For example, the empirical relation of body weight to gender does not depend on the gender distribution in the study base."* [[5], p. 56]

It is generally accepted that in order to produce results that are generalisable, studies should exhibit sufficient variability in the determinant and modifiers to be studied and a limited range for confounders [4,5]. Nevertheless the possibility of bias cannot be excluded, and empirical data on how exposure-outcome relationships might vary according to the degree of nonresponse are lacking.

Nonresponse is a form of self-selection. Selection solely by the exposure or outcome variable does not bias the estimates of ORs in logistic regression [14,30,31] and selection solely on the basis of covariates in the logistic regression also leads to unbiased ORs. Although evidence from simulation supports the principle of generalisability [12], specific scenarios may result in significant bias if selection criteria and dependent variables are closely related [32]. In particular, biases can occur if selection depends on both the exposure and outcome [14,17].

We found that although some prevalence estimates varied between the two studies of the same population investigated here, exposure-outcome relationships did not differ materially, where the variables used were highly comparable. This was despite major differences between the studies, including varying response rates, sampling frames and modes of administration; the PHS had a smaller proportion of missing and invalid responses due to the nature of the computer assisted telephone interviewing system and it included respondents who completed the survey in languages other than English. It was not possible to definitively separate the individual effects of sampling frame, response rate and mode of administration, since response rates and aspects of study design are closely linked [33].

We were unable to locate other empirical comparisons of relative risk estimates in independent studies with divergent response rates and different study designs that were drawn from the same target population. Indirect evidence supporting our findings comes from studies that have observed consistent ORs in study respondents and non-respondents using linked data [34] and in initial cohort study participants and participants responding to a subsequent questionnaire [35]. Two studies found only small biases in relative risk estimates due to nonresponse, in cross-sectional ORs from a cohort study relating to cardiovascular disease [31], and in cohort analyses relating to reproductive outcomes [12]. One study found consistent ORs related to smoking in respondents recruited by postal survey and those recruited through postal and telephone surveys and home visits [18].

Having established the lack of any major differences attributable to response rate and study design (including sampling frame and mode of questionnaire administration), the comparison of exposure-outcome relationships containing moderately comparable variables across the two studies can be seen as illustrating the additional effect of the specific questionnaire items used. Our findings demonstrate that an apparently minor difference in the wording of questions can significantly influence measures of prevalence and estimates of risks. This emphasizes the critical importance of maintaining the consistency of survey questions if valid comparisons are to be made and is consistent with previous studies [36-38]. Although most differences attributable to question wording resulted in minor heterogeneity, highly significant heterogeneity was evident for the question on self-rated health status, where the response categories varied across the two studies. However, despite differences between questionnaire items, the observed ORs would lead to similar conclusions regarding the nature of the exposure-outcome relationships.

One shortcoming of our study is the lack of strict gold-standard measures for the study variables. The PHS has a 40% nonresponse rate and may be subject to nonresponse bias. Under ideal circumstances we could use census data; however the Australian Census includes only very limited health data. Additionally, these findings relate to two large studies with considerable variability in the factors included in our analyses. This ensured that there were substantial numbers of participants from each study in each exposure-outcome category, and allowed for adjustment for multiple factors. Although these findings support the principle of generalisability of findings from a relatively select group of participants, it remains possible that they are less applicable to smaller, less heterogeneous studies. These findings relate to cross-sectional analyses; prospective, longitudinal analyses are less prone to the potential biases investigated here, since baseline selection cannot be influenced by outcome status.

Applying weights to survey data to calculate prevalence estimates that account for the differences in probability of selection is standard practice. However, use of sampling weights is less common when calculating relative risks from cohort study data; instead adjustments are usually made to account for potential confounders. The relative risk estimates adjusting for age, sex and remoteness from the 45 and Up Study were not altered materially by further weighting. Hence weighting did not appear to be necessary when the variables used in calculating the weights were used as covariates in the analysis. Weighting is potentially important in the PHS because of the role of household size and area health service in the weighting.

## Conclusions

These findings show that broad ranges of exposure-outcome relationships estimated from two studies of the same population remained consistent regardless of the underlying response rate or mode of questionnaire administration. They provide empirical support for the basic epidemiological principle that results based on internal comparisons remain generalisable even when study subjects are drawn from a relatively select group. They emphasize the crucial importance of maintaining the consistency of question wording in order to permit comparisons between studies.

## Competing interests

The authors declare that they have no competing interests.

## Authors' contributions

NM, EB, LJ and KR participated in the study concept and design and were involved in the acquisition of data. NM and EB carried out the literature review and drafted the manuscript. All authors were involved in the analysis and interpretation of data. NM performed the statistical analysis and DS, MC and KR provided statistical advice. All authors critically revised the manuscript for important intellectual content. EB and LJ obtained funding for this study and provided study supervision. All authors read and approved the final manuscript.

## Pre-publication history

The pre-publication history for this paper can be accessed here:

http://www.biomedcentral.com/1471-2288/10/26/prepub

## Supplementary Material

Additional file 1 Questionnaire items rated as highly or moderately comparable on the 45 and Up Study and the NSW PHS and used in the analyses. Click here for file

Additional file 2 Ten crude and adjusted ORs from the 45 and Up Study (unweighted and weighted), where the exposure and outcome variables were highly comparable across the 45 and Up Study and the NSW PHS. Click here for file

Additional file 3 Ten crude and adjusted ORs from the 45 and Up Study (unweighted and weighted), where either the exposure or outcome or both variables were only moderately comparable across the 45 and Up Study and the NSW PHS. Click here for file

Additional file 4 Ten crude and adjusted ORs from the NSW PHS (unweighted and weighted), where the exposure and outcome variables were highly comparable across the 45 and Up Study and the NSW PHS. Click here for file

Additional file 5 Ten crude and adjusted ORs from the NSW PHS (unweighted and weighted), where either the exposure or outcome or both variables were only moderately comparable across the 45 and Up Study and the NSW PHS. Click here for file

Additional file 6 Weighted odds ratios by study where the exposure and outcome variables were highly comparable across the 45 and Up Study and the NSW PHS. Click here for file

Additional file 7 Weighted odds Ratios by study where either the exposure or outcome or both variables were only moderately comparable across the 45 and Up Study and the NSW PHS. Click here for file
