# Supplementary material for: Investigation of relative risk estimates from studies of the same population with contrasting response rates and designs
Source: BMC Med Res Methodol. 2010 Apr 1;10:26. doi: 10.1186/1471-2288-10-26 (PMC2868856; doi:10.1186/1471-2288-10-26)
Supplement: Additional file 1 — Questionnaire items rated as highly or moderately comparable on the 45 and Up Study and the NSW PHS and used in the analyses. [file 1471-2288-10-26-S1.DOC]

Supplementary Table 1: Questionnaire items rated as highly or moderately comparable on the 45 and Up Study and the New South Wales Population Health Survey and used in the analyses.

| **Variable** ^a^ |  | **45 and Up Study** | | |  | **NSW Population Health Survey** | | |
| --- | --- | --- | --- | --- | --- | --- | --- | --- |
|  |  | Question |  | Response |  | Question |  | Response ^b^ |
|  |  |  |  |  |  |  |  |  |
| Age |  | What is your date of birth?  What is today’s date? |  | Day/Month/Year  Day/Month/Year |  | Could you please tell me how old you are today? |  | Open response for age in years |
|  |  |  |  |  |  |  |  |  |
| Sex |  | N/A |  | Determined from the Medicare Australia database |  | Are you male or female? (Only ask if unsure) |  | Male; Female |
|  |  |  |  |  |  |  |  |  |
|  |  |  |  |  |  |  |  |  |
| Height |  | How tall are you without shoes? |  | Open response in centimetres or feet and inches |  | How tall are you without shoes? |  | Open response in centimetres or feet and inches |
|  |  |  |  |  |  |  |  |  |
| Weight |  | How much do you weigh without clothes or shoes? |  | Open response in kilograms or stone and pounds |  | How much do you weigh without clothes or shoes? |  | Open response in kilograms or stone and pounds |
|  |  |  |  |  |  |  |  |  |
| Language other than English spoken at home |  | Do you speak a language other than English at home? |  | Yes; no |  | Do you usually speak a language other than English at home? |  | Yes; no |
|  |  |  |  |  |  |  |  |  |
| Country of Birth Australia |  | In which country were you born? |  | Australia; UK; Ireland; Italy; China; Greece; New Zealand; Germany; Lebanon; Philippines; Netherlands; Vietnam; Malta; Poland; other (please specify) |  | In which country were you born? |  | Australia; other country (please specify) |
|  |  |  |  |  |  |  |  |  |
| Private Health Insurance |  | Excluding Medicare, which type of insurance do you have? |  | Private health insurance - with extras; Private health insurance - without extras; Department of Veteran Affairs white or gold card; health care concession card; none of these |  | Apart from Medicare, are you currently covered by private health insurance? |  | Yes; no |
|  |  |  |  |  |  |  |  |  |
| Fruit consumption ^c^ |  | About how many serves of fruit do you usually have each day? |  | Open response for serves per day; don’t eat fruit |  | How many serves of fruit do you usually have each day? |  | Open response for serves per day; open response for serves per week; don’t eat fruit |
|  |  |  |  |  |  |  |  |  |
| Teeth |  | How many of your own teeth do you have left? |  | None – all of my teeth are missing; 1-9 teeth left; 10-19 teeth left; 20 or more teeth left |  | Are any of your natural teeth missing? |  | Yes: have some natural teeth missing; Yes: have all natural teeth missing; No: have no natural teeth missing |
|  |  |  |  |  |  |  |  |  |
| Hypertension |  | Has a doctor EVER told you that you have: high blood pressure? |  | Cross the ‘Yes’ box or leave blank |  | Have you ever been told by a doctor or a nurse that you have high blood pressure? ^d^ |  | Yes; no |
|  |  |  |  |  |  |  |  |  |
| Diabetes |  | Has a doctor EVER told you that you have: diabetes? |  | Cross the ‘Yes’ box or leave blank |  | Have you ever been told by a doctor or at a hospital that you have diabetes? |  | Yes; no |
|  |  |  |  |  |  |  |  |  |
| Asthma |  | Has a doctor EVER told you that you have: asthma? |  | Cross the ‘Yes’ box or leave blank |  | Have you ever been told by a doctor or at a hospital that you have asthma? |  | Yes; no |
|  |  |  |  |  |  |  |  |  |
| Fallen in past 12 months |  | During the past 12 months, how many times have you fallen to the ground? (Put “0” if you haven’t fallen in this time) |  | Open response |  | In the last 12 months have you had a fall? ^e^ |  | Yes; no |
|  |  |  |  |  |  |  |  |  |
| Breast screening in past 2 years |  | Have you ever been for a breast screening mammogram? If yes, what year did you have your last mammogram? |  | Yes; no.  Open response for year |  | A mammogram is an X-ray taken of the breasts by a machine that presses against the breast while the picture is taken. It is a means of detecting breast cancer in the early stages. Have you ever had a mammogram? When did you last have a mammogram? ^f^ |  | Yes; no  Less than 1 year ago; 1 year to less than 2 years ago; 2 years to less than 3 years ago; 3 years to less than 4 years ago; 4 years to less than 5 years ago; 5 or more years ago. |
|  |  |  |  |  |  |  |  |  |
| Hysterectomy |  | Have you ever had any of the following operations? hysterectomy |  | Cross the ‘Yes’ box or leave blank |  | A hysterectomy is an operation in which a woman's uterus (or womb) is removed. Have you ever had a hysterectomy? ^g^ |  | Yes; no |
|  |  |  |  |  |  |  |  |  |
| Psychological Distress |  | Asked the K10 10-item questionnaire that measures the level of psychological distress in the most recent 4-week period. |  | The ten questions and responses were identical (word-for-word) across the two surveys |  | Asked the K10 10-item questionnaire that measures the level of psychological distress in the most recent 4-week period. |  | The ten questions and responses were identical (word-for-word) across the two surveys |
|  |  |  |  |  |  |  |  |  |
|  |  |  |  |  |  |  |  |  |
| Educational Attainment |  | What is the highest qualification you have completed? |  | No school certificate or other qualifications; school or intermediate certificate (or equivalent); higher school or leaving certificate (or equivalent); trade/apprenticeship (e.g. hairdresser, chef); certificate/diploma (e.g. child care, technician); university degree or higher |  | What is the level of the highest qualification you have completed? |  | Completed school certificate or intermediate or year 10 or 4^th^ form; completed higher school certificate or leaving or year 12 or 6th form; TAFE certificate or diploma; university, college of advanced education, or some other tertiary institute degree or higher; other (specify); completed primary school; completed years 7 to 9 |
|  |  |  |  |  |  |  |  |  |
| Pre-tax Income |  | What is your usual yearly HOUSEHOLD income before tax, from all other sources? (please include benefits, pensions, superannuation etc) |  | Less than $5,000; $5,000-$9,999: $10,000-$19,999; $20,000-$29,999; $30,000-$39,999; $40,000-$49,000; $50,000-$69,000; $70,000 or more per year |  | What is your annual household income before tax? Would it be: |  | Less than $20,000; $20,000-$40,000; $40,000- $60,000; $60,000-$80,000; more than $80,000 |
|  |  |  |  |  |  |  |  |  |
| Current regular smoker |  | Are you a regular smoker now? |  | Yes; no |  | Which of the following best describes your smoking status? This includes cigarettes, cigars and pipes. |  | I smoke daily; I smoke occasionally; I don't smoke now, but I used to; I've tried it a few times but never smoked regularly; I've never smoked |
|  |  |  |  |  |  |  |  |  |
| Bowel Screening in past 5 years |  | Have you ever been screened for colorectal (bowel) cancer? If Yes, please indicate which test(s) you had:  What year did you have the most recent  one of these tests? (e.g. 2005) |  | Yes; no  - faecal occult blood test (test for blood in the stool/faeces)  - sigmoidoscopy (a tube is used to examine the lower bowel: this is usually done in a doctor’s office without pain relief)  - colonoscopy (a long tube is used to examine the whole large bowel: you would usually have to have an enema or drink large amounts  of special liquid to prepare the bowel for this)  Open response for year |  | Bowel cancer is a  common cancer  which, if found, can  be treated at an  early stage. Bowel  cancer may be  detected by using  several different  types of  investigations.  Have you ever had:  When did you have  your last x-ray/ colonoscopy/ faeces sample examined? ^h^ |  | An X-ray of the  Bowel (Yes or no);  A colonoscopy (Yes or no);  Sample of faeces  examined for bowel cancer (Yes or no); None of the above (Yes or no)  Within the last 12 months; 12 months to 5 years; More than 5 years ago; Never had a bowel x-ray/ test with a tube like  instrument/ faeces sample examined |
|  |  |  |  |  |  |  |  |  |
| Self-Reported Health Status |  | Overall, how would you rate your overall health? |  | Excellent; very good; good; fair; poor |  | Overall, how would you rate your health during the past 4 weeks? |  | Excellent; very good; good; fair; poor; very poor |
|  |  |  |  |  |  |  |  |  |

NSW=New South Wales

^a^ Variables above the line-break are highly comparable across the two surveys. Variables below the line-break are moderately comparable but similar across the two surveys

^b^ All questions on the NSW Population Health Survey had the response options of ‘Don’t Know’ and ‘Refused’

^c^ Definition of one serve of fruit identical on both surveys

^d^ This question was asked on the 2007 NSW Population Health Survey only

^e^ This question was asked of participants aged less than 60 years only

^f^ This question was only asked on the 2006 NSW Population Health Survey and for participants aged less than 80 years only

^g^ This question was only asked on the 2006 NSW Population Health Survey and for participants aged less than 70 years only

^h^ This question was only asked on the 2007 NSW Population Health Survey and for participants aged over 50 years only
