# Supplementary material for: Investigation of relative risk estimates from studies of the same population with contrasting response rates and designs
Source: BMC Med Res Methodol. 2010 Apr 1;10:26. doi: 10.1186/1471-2288-10-26 (PMC2868856; doi:10.1186/1471-2288-10-26)
Supplement: Additional file 2 — Ten crude and adjusted ORs from the 45 and Up Study (unweighted and weighted), where the exposure and outcome variables were highly comparable across the 45 and Up Study and the NSW PHS. [file 1471-2288-10-26-S2.DOC]

Supplementary Table 2: Ten crude and adjusted odds ratios from the 45 and Up Study (unweighted and weighted ^a^), where the exposure and outcome variables were highly comparable across the 45 and Up Study and the New South Wales Population Health Survey.

| **Outcome** | **Exposure** | **Crude Odds Ratio**  **(95% C.I.)** | | **Odds Ratio adjusting for age, sex and remoteness (95% C.I.)** | |
| --- | --- | --- | --- | --- | --- |
|  |  | Unweighted | Weighted | Unweighted | Weighted |
| Diabetes | Body Mass Index |  |  |  |  |
|  | Underweight | 1.01 (0.79, 1.29) | 1.06 (0.80, 1.40) | 0.95 (0.74, 1.21) | 0.97 (0.73, 1.29) |
|  | Normal Range ^b^ | 1 | 1 | 1 | 1 |
|  | Overweight | 1.71 (1.61, 1.82) | 1.64 (1.53, 1.77) | 1.66 (1.56, 1.76) | 1.60 (1.49, 1.72) |
|  | Obese | 3.73 (3.51, 3.97) | 3.78 (3.52, 4.07) | 4.17 (3.92, 4.44) | 4.13 (3.84, 4.45) |
| Hypertension | Body Mass Index |  |  |  |  |
|  | Underweight | 0.69 (0.59, 0.81) | 0.64 (0.53, 0.78) | 0.75 (0.63, 0.88) | 0.72 (0.59, 0.88) |
|  | Normal Range ^b^ | 1 | 1 | 1 | 1 |
|  | Overweight | 1.80 (1.74, 1.87) | 1.88 (1.80, 1.96) | 1.55 (1.49, 1.61) | 1.57 (1.50, 1.65) |
|  | Obese | 2.49 (2.39, 2.59) | 2.74 (2.61, 2.88) | 2.72 (2.61, 2.85) | 2.80 (2.66, 2.95) |
| Fallen in past | Body Mass Index |  |  |  |  |
| 12 months | Underweight | 1.49 (1.28, 1.74) | 1.58 (1.32, 1.89) | 1.20 (1.02, 1.41) | 1.20 (0.99, 1.45) |
|  | Normal Range ^b^ | 1 | 1 | 1 | 1 |
|  | Overweight | 0.91 (0.87, 0.96) | 0.89 (0.83, 0.94) | 1.06 (1.00, 1.11) | 1.05 (0.99, 1.12) |
|  | Obese | 1.23 (1.16, 1.31) | 1.21 (1.13, 1.30) | 1.49 (1.40, 1.59) | 1.50 (1.39, 1.61) |
| Fallen in past | Age |  |  |  |  |
| 12 months | 60-64 years ^b^ | 1 | 1 | 1 | 1 |
|  | 65-69 years | 0.98 (0.92, 1.05) | 1.03 (0.96, 1.11) | 1.00 (0.93, 1.07) | 1.03 (0.95, 1.11) |
|  | 70-74 years | 1.22 (1.14, 1.31) | 1.33 (1.22, 1.44) | 1.26 (1.18, 1.35) | 1.31 (1.21, 1.42) |
|  | 75-79 years | 1.61 (1.50, 1.73) | 1.83 (1.68, 1.98) | 1.68 (1.57, 1.81) | 1.79 (1.65, 1.95) |
|  | 80-84 years | 2.04 (1.91, 2.19) | 2.33 (2.16, 2.52) | 2.24 (2.09, 2.40) | 2.25 (2.08, 2.43) |
|  | ≥ 85 years | 3.12 (2.87, 3.40) | 3.56 (3.24, 3.92) | 3.30 (3.03, 3.59) | 3.30 (3.00, 3.63) |
| High or | Age |  |  |  |  |
| Very High | 45-49 years ^b^ | 1 | 1 | 1 | 1 |
| Psychological | 50-54 years | 0.91 (0.84, 0.99) | 0.94 (0.85, 1.03) | 0.92 (0.85, 0.99) | 0.94 (0.86, 1.03) |
| Distress | 55-59 years | 0.79 (0.73, 0.86) | 0.82 (0.75, 0.90) | 0.80 (0.74, 0.87) | 0.82 (0.75, 0.90) |
|  | 60-64 years | 0.61 (0.56, 0.67) | 0.64 (0.58, 0.71) | 0.62 (0.56, 0.68) | 0.64 (0.58, 0.71) |
|  | 65-69 years | 0.50 (0.45, 0.55) | 0.52 (0.46, 0.58) | 0.50 (0.46, 0.56) | 0.51 (0.46, 0.58) |
|  | 70-74 years | 0.42 (0.38, 0.48) | 0.48 (0.42, 0.55) | 0.43 (0.38, 0.49) | 0.48 (0.42, 0.55) |
|  | 75-79 years | 0.40 (0.35, 0.46) | 0.46 (0.39, 0.54) | 0.41 (0.36, 0.48) | 0.46 (0.39, 0.54) |
|  | 80-84 years | 0.55 (0.48, 0.62) | 0.62 (0.53, 0.72) | 0.57 (0.50, 0.65) | 0.61 (0.53, 0.71) |
|  | ≥ 85 years | 0.79 (0.67, 0.94) | 0.97 (0.81, 1.17) | 0.83 (0.70, 0.97) | 0.95 (0.79, 1.14) |
| High or | Remoteness (ARIA+) |  |  |  |  |
| Very High | Major City ^b^ | 1 | 1 | 1 | 1 |
| Psychological | Inner Regional | 1.06 (1.00, 1.12) | 1.04 (0.98, 1.10) | 1.06 (1.00, 1.12) | 1.06 (1.00, 1.12) |
| Distress | Outer Regional | 1.05 (0.98, 1.12) | 1.03 (0.96, 1.11) | 1.05 (0.98, 1.13) | 1.06 (0.98, 1.14) |
|  | Remote | 1.04 (0.87, 1.25) | 1.06 (0.87, 1.28) | 1.02 (0.85, 1.22) | 1.07 (0.89, 1.30) |
| Asthma | Remoteness (ARIA+) |  |  |  |  |
|  | Major City ^b^ | 1 | 1 | 1 | 1 |
|  | Inner Regional | 1.12 (1.06 1.18) | 1.09 (1.03, 1.15) | 1.09 (1.03, 1.15) | 1.09 (1.03, 1.16) |
|  | Outer Regional | 1.18 (1.10 1.26) | 1.12 (1.05, 1.20) | 1.15 (1.08, 1.23) | 1.13 (1.06, 1.21) |
|  | Remote | 1.52 (1.29 1.79) | 1.43 (1.20, 1.70) | 1.47 (1.25, 1.74) | 1.45 (1.22, 1.73) |
| 2 or more | Remoteness (ARIA+) |  |  |  |  |
| serves | Major City ^b^ | 1 | 1 | 1 | 1 |
| of fruit | Inner Regional | 0.95 (0.92, 0.98) | 0.94 (0.91, 0.97) | 0.93 (0.90, 0.95) | 0.93 (0.90, 0.96) |
| per day | Outer Regional | 0.88 (0.85, 0.92) | 0.86 (0.83, 0.89) | 0.85 (0.82, 0.89) | 0.85 (0.82, 0.89) |
|  | Remote | 0.69 (0.63, 0.75) | 0.65 (0.59, 0.71) | 0.67 (0.61, 0.73) | 0.66 (0.60, 0.73) |
| Breast | Country of Birth |  |  |  |  |
| Screened in | Australia ^b^ | 1 | 1 | 1 | 1 |
| past 2 years | Not Australia | 0.89 (0.84 0.93) | 0.89 (0.84, 0.94) | 0.81 (0.77, 0.85) | 0.82 (0.77, 0.88) |
|  |  |  |  |  |  |
| Hysterectomy | Private Health Insurance |  |  |  |  |
|  | No ^b^ | 1 | 1 | 1 | 1 |
|  | Yes | 0.89 (0.85, 0.93) | 0.89 (0.84, 0.94) | 0.94 (0.89, 0.99) | 0.94 (0.89, 1.00) |

ARIA+, Accessibility Remoteness Index of Australia; CI, Confidence Interval;

^a^ Weighted by age, sex and remoteness

^b^ Reference Category
