# Supplementary material for: Investigation of relative risk estimates from studies of the same population with contrasting response rates and designs
Source: BMC Med Res Methodol. 2010 Apr 1;10:26. doi: 10.1186/1471-2288-10-26 (PMC2868856; doi:10.1186/1471-2288-10-26)
Supplement: Additional file 5 — Ten crude and adjusted ORs from the NSW PHS (unweighted and weighted), where either the exposure or outcome or both variables were only moderately comparable across the 45 and Up Study and the NSW PHS. [file 1471-2288-10-26-S5.DOC]

Supplementary Table 5: Ten crude and adjusted odds ratios from the NSW PHS (unweighted and weighted ^a^), where either the exposure or outcome or both variables were only moderately comparable across the 45 and Up Study and the NSW PHS.

| **Outcome** | **Exposure** | **Crude Odds Ratio**  **(95% C.I.)** | | **Odds Ratio adjusting for age, sex and remoteness (95% C.I.)** | |
| --- | --- | --- | --- | --- | --- |
|  |  | Unweighted | Weighted | Unweighted | Weighted |
| Current | All natural teeth missing |  |  |  |  |
| Smoker | No ^b^ | 1 | 1 | 1 | 1 |
|  | Yes | 1.24 (1.03, 1.50) | 1.19 (0.94, 1.49) | 2.47 (2.00, 3.05) | 2.54 (1.94, 3.31) |
|  |  |  |  |  |  |
| Current | Educational Attainment |  |  |  |  |
| Smoker | No School Certificate ^b^ | 1 | 1 | 1 | 1 |
|  | School Certificate | 0.98 (0.81, 1.18) | 0.97 (0.75, 1.25) | 0.83 (0.68, 1.01) | 0.85 (0.66, 1.11) |
|  | Diploma/Trade/Certificate | 0.93 (0.77, 1.11) | 0.92 (0.71, 1.18) | 0.62 (0.51, 0.75) | 0.65 (0.50, 0.85) |
|  | Higher School Certificate | 0.95 (0.75, 1.20) | 1.02 (0.75, 1.38) | 0.70 (0.55, 0.89) | 0.75 (0.54, 1.03) |
|  | Tertiary qualification | 0.57 (0.46, 0.70) | 0.49 (0.37, 0.65) | 0.34 (0.27, 0.42) | 0.30 (0.22, 0.41) |
|  |  |  |  |  |  |
| Obesity | Educational Attainment |  |  |  |  |
|  | No School Certificate ^b^ | 1 | 1 | 1 | 1 |
|  | School Certificate | 0.94 (0.81, 1.10) | 1.04 (0.85, 1.27) | 0.85 (0.73, 1.00) | 0.95 (0.78, 1.17) |
|  | Diploma/Trade/Certificate | 0.81 (0.70, 0.94) | 0.88 (0.73, 1.07) | 0.70 (0.60, 0.82) | 0.77 (0.63, 0.94) |
|  | Higher School Certificate | 0.74 (0.61, 0.89) | 0.77 (0.60, 0.99) | 0.66 (0.54, 0.80) | 0.68 (0.53, 0.88) |
|  | Tertiary qualification | 0.57 (0.48, 0.67) | 0.59 (0.48, 0.74) | 0.47 (0.39, 0.56) | 0.49 (0.39, 0.62) |
|  |  |  |  |  |  |
| Bowel | Smoking Status |  |  |  |  |
| Screened | Non-current Smoker ^b^ | 1 | 1 | 1 | 1 |
| in past 5 years | Current Smoker | 0.54 (0.42, 0.69) | 0.55 (0.39, 0.77) | 0.59 (0.46, 0.76) | 0.57 (0.40, 0.81) |
|  |  |  |  |  |  |
| Bowel | Fruit consumption |  |  |  |  |
| Screened | Don't eat fruit | 0.84 (0.60, 1.18) | 0.80 (0.51, 1.25) | 0.88 (0.63, 1.24) | 0.82 (0.52, 1.29) |
| in past 5 years | < 2 serves per day | 0.93 (0.78, 1.11) | 0.95 (0.76, 1.18) | 0.94 (0.79, 1.12) | 0.96 (0.77, 1.20) |
|  | 2+ serves per day ^b^ | 1 | 1 | 1 | 1 |
|  |  |  |  |  |  |
| High or | Self-rated Health Status |  |  |  |  |
| Very High | Excellent ^b^ | 1 | 1 | 1 | 1 |
| Psychological | Very Good | 1.90 (1.33, 2.70) | 2.17 (1.35, 3.50) | 1.96 (1.38, 2.79) | 2.23 (1.39, 3.60) |
| Distress | Good | 4.12 (2.97, 5.72) | 4.72 (3.02, 7.37) | 4.40 (3.16, 6.12) | 4.91 (3.14, 7.67) |
|  | Fair | 8.62 (6.21, 11.97) | 7.83 (5.04, 12.18) | 10.06 (7.22, 14.02) | 9.20 (5.89, 14.38) |
|  | Poor | 24.37 (17.44, 34.06) | 26.46 (16.83, 41.61) | 27.57 (19.63, 38.72) | 31.07 (19.63, 49.18) |
|  |  |  |  |  |  |
| High or | Pre-tax Income |  |  |  |  |
| Very High | <$20,000 p.a. | 1.51 (1.27, 1.78) | 1.53 (1.21, 1.94) | 1.77 (1.49, 2.12) | 1.80 (1.41, 2.30) |
| Psychological | $20,000-$39,999 p.a. ^b^ | 1 | 1 | 1 | 1 |
| Distress | $40,000+ p.a. | 0.71 (0.58, 0.85) | 0.66 (0.50, 0.85) | 0.46 (0.38, 0.56) | 0.44 (0.33, 0.59) |
|  |  |  |  |  |  |
| Private Health | Pre-tax Income |  |  |  |  |
| Insurance | <$20,000 p.a. | 0.38 (0.34, 0.41) | 0.43 (0.38, 0.48) | 0.35 (0.32, 0.39) | 0.39 (0.35, 0.45) |
|  | $20,000-$39,999 p.a. ^b^ | 1 | 1 | 1 | 1 |
|  | $40,000+ p.a. | 2.97 (2.69, 3.27) | 3.31 (2.92, 3.77) | 3.60 (3.23, 4.00) | 4.09 (3.54, 4.72) |
|  |  |  |  |  |  |
| Pre-tax | Remoteness (ARIA+) |  |  |  |  |
| income | Major City ^b^ | 1 | 1 | 1 | 1 |
| ≥ $40,000 | Inner Regional | 0.75 (0.69, 0.82) | 0.70 (0.63, 0.78) | 0.68 (0.62, 0.75) | 0.64 (0.57, 0.73) |
| per annum | Outer Regional | 0.58 (0.53, 0.64) | 0.52 (0.46, 0.59) | 0.50 (0.45, 0.56) | 0.48 (0.42, 0.55) |
|  | Remote | 0.67 (0.54, 0.83) | 0.61 (0.47, 0.79) | 0.56 (0.44, 0.71) | 0.55 (0.41, 0.73) |
|  |  |  |  |  |  |
| Fallen in past | Self-rated Health Status |  |  |  |  |
| 12 months | Excellent ^b^ | 1 | 1 | 1 | 1 |
|  | Very Good | 1.26 (0.97, 1.65) | 1.57 (1.15, 2.16) | 1.17 (0.89, 1.54) | 1.43 (1.04, 1.96) |
|  | Good | 1.50 (1.16, 1.94) | 1.73 (1.27, 2.35) | 1.44 (1.10, 1.87) | 1.60 (1.17, 2.18) |
|  | Fair | 2.17 (1.65, 2.84) | 2.46 (1.78, 3.40) | 1.94 (1.47, 2.56) | 2.10 (1.51, 2.93) |
|  | Poor | 3.23 (2.37, 4.41) | 3.89 (2.66, 5.68) | 2.88 (2.09, 3.95) | 3.38 (2.29, 4.99) |

ARIA+, Accessibility Remoteness Index of Australia; CI, Confidence Interval; NSW, New South Wales; p.a, per annum; PHS, Population Health Survey

^a^ Weighted by area health service, probability of selection in the household and the number of residential connections to the house

^b^ Reference Category
