# Supplementary figures and images for: Investigation of relative risk estimates from studies of the same population with contrasting response rates and designs
Source: BMC Med Res Methodol. 2010 Apr 1;10:26. doi: 10.1186/1471-2288-10-26 (PMC2868856; doi:10.1186/1471-2288-10-26)

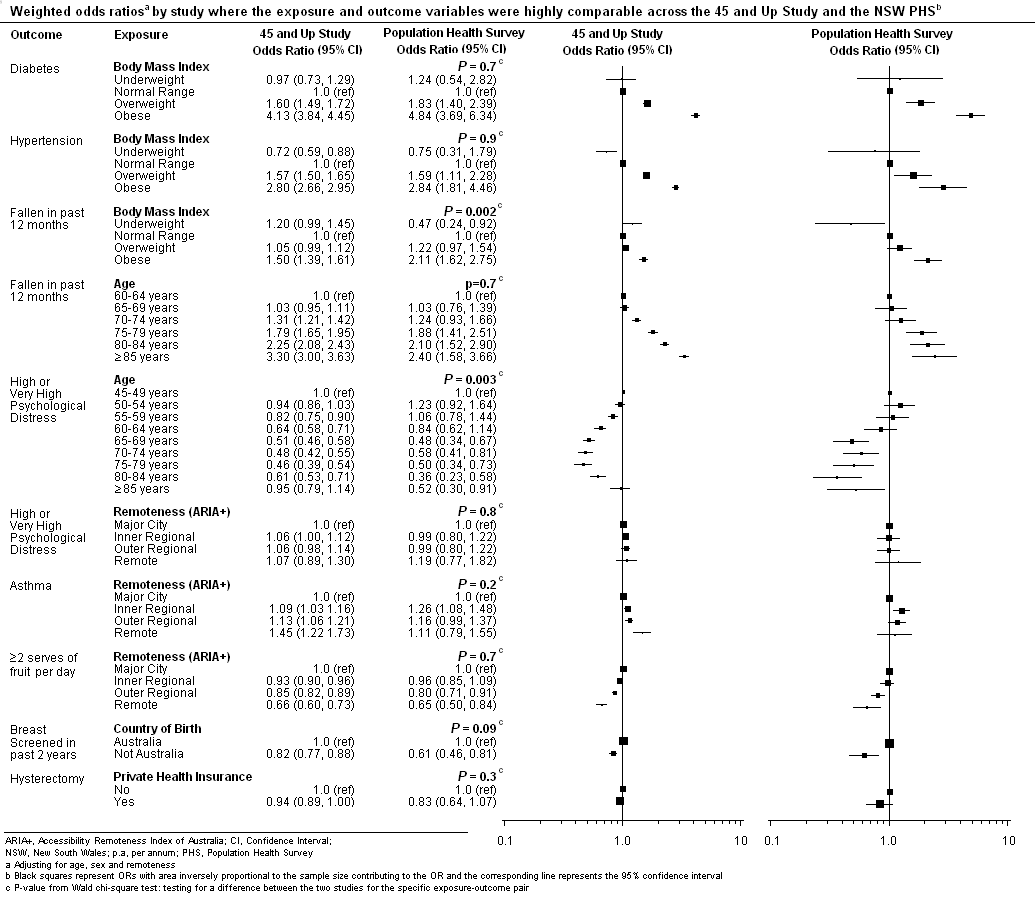

Supplement: Additional file 6 — Weighted odds ratios by study where the exposure and outcome variables were highly comparable across the 45 and Up Study and the NSW PHS. [file 1471-2288-10-26-S6.BMP]

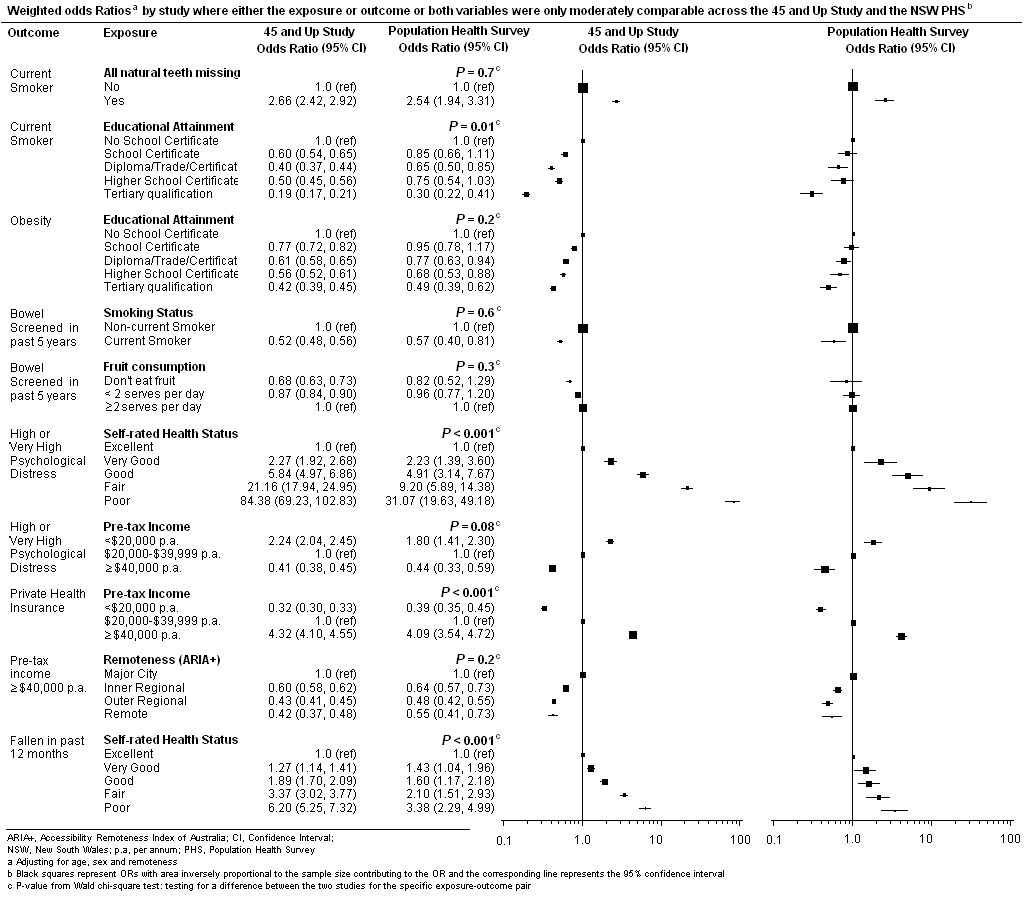

Supplement: Additional file 7 — Weighted odds Ratios by study where either the exposure or outcome or both variables were only moderately comparable across the 45 and Up Study and the NSW PHS. [file 1471-2288-10-26-S7.BMP]
